# The Interplay of Vitamin D Deficiency and Cellular Senescence in The Pathogenesis of Obesity-Related Co-Morbidities

**DOI:** 10.3390/nu13114127

**Published:** 2021-11-17

**Authors:** Abdulhadi Bima, Basmah Eldakhakhny, Dina Nuwaylati, Abrar Alnami, Mohammed Ajabnoor, Ayman Elsamanoudy

**Affiliations:** 1 Department of Clinical Biochemistry, Faculty of Medicine, King AbdulAziz University, Jeddah 21465, Saudi Arabia; hadibima@hotmail.com (A.B.); beldakhakhny@kau.edu.sa (B.E.); Abraralnami@outlook.com (A.A.); ma_ajabnoor@yahoo.com (M.A.); 2 Department of Clinical Biochemistry, Faculty of Medicine, University of Jeddah, Jeddah 21959, Saudi Arabia; dina.nuwaylati@gmail.com; 3 Medical Biochemistry and Molecular Biology, Faculty of Medicine, Mansoura University, Mansoura 35516, Egypt

**Keywords:** vitamin D deficiency, cellular senescence, obesity, non-alcoholic fatty liver disease, subclinical atherosclerosis

## Abstract

This scoping review aims to clarify the interplay between obesity, vitamin D deficiency, cellular senescence, and obesity-related metabolic consequences, mainly subclinical atherosclerosis, and non-alcoholic fatty liver disease (NAFLD). Obesity is a significant global health problem that involves cellular, environmental, behavioral, and genetic elements. The fundamental cause of obesity throughout all life stages is an energy imbalance, and its consequences are countless and, foremost, very common. Obesity has been comprehensively studied in the literature given its association with low serum vitamin D, with many proposed mechanisms linking the two conditions. Moreover, markers of exaggerated cellular senescence have been proven to accumulate in obese individuals. Subclinical atherosclerosis initiates an early stage that ends in serious cardiac events, and obesity, low vitamin D, and senescent cells largely contribute to its associated chronic low-grade inflammation. Furthermore, NAFLD signifies the hepatic manifestation of metabolic syndrome, and studies have highlighted the important role of obesity, vitamin D deficiency, and cellular senescence in its development. Therefore, we outlined the most important mechanisms tying these conditions to one another.

## 1. Introduction

Years of research have revealed the association of three conditions with the development of subclinical atherosclerosis and non-alcoholic fatty liver disease (NAFLD): obesity, vitamin D deficiency (VDD), and cellular senescence. Obesity is a serious worldwide chronic health problem for all ages [1]. Cellular senescence induces a cell-cycle arrest and a pro-inflammatory reaction, both of which promote aging and age-related diseases, and its exacerbation was seen with obesity in many studies [2]. VDD was reported to be induced by obesity among all age groups and in both genders [1,3]. Numerous genetic studies explored that excessive adiposity causes a reduction in circulating 25-hydroxyvitamin D (25(OH)D), the main reflector of vitamin D status [1,3]. VDD in obese people usually has no direct consequence but may affect many organs at the subclinical level and predispose them to a state of improper metabolism [4]. On the other hand, the effects of vitamin D supplementation on obese individuals do not largely extend beyond ameliorating the detrimental consequences of various obesity-induced cardiometabolic disorders, and no evidence proves a protective effect of vitamin D against obesity [1,3]. Obesity promotes cardiovascular diseases through vascular remodeling, which induces subclinical atherosclerosis that finally ends in fatal cardiac events [5]. Additionally, a wide spectrum of liver disorders is associated with obesity, including NAFLD, which is emerging as a serious health problem due to its potential to progress to end-stage liver cirrhosis [6]. This scoping review aims to discuss obesity, VDD, and cellular senescence and the possible mechanisms by which they contribute to the occurrence and progression of subclinical atherosclerosis and NAFLD.

## 2. Materials and Methods

We reviewed published articles that discussed obesity-related disorders, particularly subclinical atherosclerosis and NAFLD, and their correlation with VDD and cellular senescence. Databases used for extracting articles were PubMed, PubMed Central, Cochrane Database of Systematic Reviews, MEDLINE, MedlinePlus, Google Scholar database, and WHO reports, published from 2000 to 2021. The main search terms used were obesity, obesity-related cardiometabolic diseases, vitamin D deficiency, non-alcoholic fatty liver disease, cellular senescence, and subclinical atherosclerosis. In this scoping review, we emphasized the interplay between obesity, VDD, and cellular senescence as pathophysiological mechanisms that play a role in subclinical atherosclerosis and NAFLD.

## 3. Obesity and Its Associated Alteration in Adipose Tissue Microenvironment

Obesity is defined as the harmful and exaggerated fat accumulation in the body [7]. Typically, body mass index (BMI) is relied on as a biomarker for obesity [8], and a BMI of ≥30 kg/m^2^ describes obesity according to the World Health Organization [7]. The majority of individuals categorized as obese based on their BMIs do progress to obesity-associated cardiometabolic diseases. On the other hand, some may stay healthy for longer periods during their lifetime and are considered to have metabolically healthy obesity (MHO) [8,9,10]. People with MHO do not have any other cardiovascular risk factors [9]; however, they are still at risk for developing cardiometabolic complications later in life due to long-term exposure to both general and abdominal adiposity [8,10,11]. Since BMI does not reflect abdominal adiposity, it is insufficient to capture the cardiometabolic risk among those with MHO; therefore, waist circumference has been recommended as an additive tool to reflect the cardiometabolic risk, especially among those with MHO, and it has been associated with both all-cause and cardiovascular mortality [8,9,11]. A waist circumference of ≥88 cm for women and ≥102 cm for men defines abdominal adiposity [8]. 

Obesity is a major risk factor for several long-standing chronic illnesses that have serious consequences, such as atherosclerotic cardiovascular diseases (ASCVD), diabetes, NAFLD, and certain types of cancer [7,12,13]. The persistent rise in obesity rates is a major driver of the burden that accompanies these conditions [12]. Grasping the fundamentals of the mechanisms underlying obesity-related disorders necessitates an understanding of obesity-induced remodeling of the adipose tissue (AT) microenvironment and the alteration of its cellular components. 

AT resembles an endocrine organ in terms of its behavior [14]. It can produce various bioactive cytokines, named adipokines, some of which are anti-inflammatory, such as adiponectin [14,15]. AT also produces pro-inflammatory adipokines, and their expression is enhanced by obesity, such as leptin, TNFα, and interleukin-6 (IL-6) [14,15]. This leads to an imbalance between anti and pro-inflammatory molecules, precipitating a state of sustained low-grade inflammation, promoting metabolic dysfunction and cardiovascular disease [14,15].

Obesity also results in the accumulation of progenitors of adipocytes in the AT, which leads to hyperplasia, and eventually hypertrophy of adipocytes from storing excess triglycerides (TAGs) [14,15]. These changes render AT to be dysfunctional in the long term from undergoing necrosis or apoptosis, as well as the recruitment of classically activated macrophages (M1) and other pro-inflammatory factors [14,15]. Conventionally, the M1 class of macrophages generates the pro-inflammatory tumor necrosis factor (TNF-α) and reactive oxygen species (ROS) and are therefore accompanied by tissue damage and inflammation [14,15]. Furthermore, the accumulation of macrophages in AT promotes IR through immunologically mediated mechanisms [14]. Energy in the body is stored in the adipose tissue, and when excess fat accumulates, adipocytes release free fatty acids (FFAs) into the circulation, which further impairs insulin sensitivity [16].

Normally, lean fat expresses numerous CD4+ regulatory T cells and TH2-polarized cells, both of which possess anti-inflammatory properties, maintain insulin sensitivity, and preserve AT homeostasis [14,15]. On the other hand, hypertrophied AT accumulates both CD8+ effector T cells and CD4+ TH1 cells, which are key promoters of M1 activation that ultimately lead to inflammation and ends in IR [14,15]. The AT remodeling explained, collectively with other processes such as impaired vascularization and endothelial cell activation in AT, can promote chronic low-grade inflammation, which is a vital factor in the progression to all metabolic dysfunctions arising from obesity [15].

## 4. Vitamin D Overview

The fat-soluble vitamin D exists in two primary forms, D2 (ergocalciferol) and D3 (cholecalciferol) [17,18]. Vitamin D2 is obtained exogenously, while the latter is supplied by dermal synthesis; when ultraviolet B radiation (UVB) hits the skin, vitamin D3 is produced [17,18,19]. Owing to the limited food types containing vitamin D2, dermal synthesis accounts for the majority of vitamin D in the body [17,18]. Both forms undergo two enzymatic hydroxylation reactions for activation [17,18]. The first is the hepatic hydroxylation by 25-hydroxylase forming 25-hydroxyvitamin D (25(OH)D), and the second is the renal hydroxylation of 25(OH)D by 1α-hydroxylase producing the biologically active calcitriol (1,25-dihydroxyvitamin D/1,25(OH)2D) [17,18]. 1α-hydroxylase has been shown to be expressed in other extra-renal tissues as well, but with a lesser significance [17]. 25(OH)D, the precursor of calcitriol, is the major circulating form of vitamin D [17,18].

Both 25(OH)D and calcitriol are carried out in the circulation bound to a carrier protein, vitamin D binding protein (DBP), which carries more than 80% of circulating vitamin D [18,19,20]. DBP is a highly polymorphic hepatic-derived protein that has various functions in several biological tissues, and not only those related to vitamin D transport, but it plays a major role in the regulation of circulating vitamin D levels [19,20]. However, the regulation of its own production is not influenced by vitamin D, yet estrogen levels, certain cytokines, parathyroid hormone, and dexamethasone administration all influence DBP production [19,20].

In healthy subjects, free (unbound) vitamin D levels are highly correlated with total (bound and unbound) levels; free vitamin D comprises only 0.02–0.09% of total vitamin D. This relationship is usually maintained with normal unaltered DBP [19]. While in cases causing any disturbance in the DBP levels, or its affinity to vitamin D metabolites, free to total vitamin D levels may no longer be maintained, which may result in deficient vitamin D [19]. Nevertheless, not all conditions with VDD are associated with altered DBP [19,20]. For instance, obesity with reduced both free and total vitamin D levels has normal concentrations and functions of DBP [19]. The exact mechanisms underlying these observations are unknown [19].

### 4.1. Vitamin D deficiency

The adequacy of vitamin D is assessed by 25(OH)D measurements as it indicates dietary vitamin D as well as the amount produced by dermal synthesis [21]. The cutoff levels determining vitamin D deficiency (VDD), or “insufficiency” as it is referred to sometimes, are inconsistent among researchers and different international guidelines. The Institute of Medicine (IOM) defines serum 25(OH)D <12 ng/mL as deficient and places a risk in relation to bone health, while levels between 12 and 20 ng/mL is considered as inadequate for both bone and overall health, >20 ng/mL as adequate (sufficient) for both bone and overall health, and levels >50 ng/mL is a reason for concern as it was suggested to be associated with adverse effects [17]. On the other hand, the Endocrine Society Clinical Practice Guidelines define 25(OH)D <20 ng/mL as deficient and 21–29 ng/mL as insufficient [21]. Measuring the biologically active calcitriol (1,25(OH)2D) is not recommended as it is a poor indicator of vitamin D status because of its short half-life [22]. Additionally, it is subject to regulation by serum parathyroid hormone (PTH) levels; in cases of VDD, renal production of calcitriol is enhanced by PTH, while serum 25(OH)D levels would be reduced [22].

Severe VDD states have been reported worldwide with high variability based on age groups and ethnicity. For instance, 25(OH)D levels of <12 ng/mL were estimated to be 5–8% in the US and Canada and 13% in Europe, while levels of <20 ng/mL were reported to be 24%, 37%, and 40% in the same countries, respectively [23]. Numerous other countries show high VDD rates; for example, more than 20% of the populations in Tunisia, India, and Pakistan have 25(OH)D levels <12 ng/mL [23]. VDD is even more prevalent in the Middle East, such as Saudi Arabia, where more than 60% of the population show 25(O)HD levels of <20 ng/mL [24].

VDD has been shown to be associated with multiple health outcomes, such as rickets, fractures, osteoporosis, cancers, hypertension, autoimmune disorders, and Alzheimer’s disease [25,26]. Accordingly, the causes of VDD will be highlighted in the following section.

### 4.2. Causes of VDD

The main cause of VDD is the lack of sufficient, yet balanced, sun exposure [25], especially with lifestyle advances and predominant indoor activities [27]. Similarly, topical sunscreens absorb nearly all UVB radiation, which decreases dermal vitamin D synthesis by 99% [25,27]. Therefore, dietary intake of vitamin D is necessary, but foods naturally containing vitamin D are very limited, such as liver, fish, eggs, mushrooms, and dairy products [27,28], and those fortified with vitamin D often do not fulfill vitamin D requirements for both children and adults [25]. Veganism was believed to increase the risk of VDD, yet some studies have shown that serum levels of 25(OHD) is multifactorial, with other aspects having a greater impact on 25(OH)D than diet, such as vitamin D supplements intake, the intensity of sun exposure, and ethnicity [28]. Additionally, vegetarians tend to have a higher rate of vitamin D supplementation than omnivores, which helps them compensate for VDD states [29].

Gastrointestinal risk factors also contribute to VDD [30]. Absorption of vitamin D occurs in the gut, besides what usually takes place in the skin. Hence, several conditions associated with a state of malabsorption, such as short bowel syndrome, pancreatitis, Celiac disease, Crohn’s disease, and bariatric surgeries all reduce 25(OH)D [30,31]. Nevertheless, the effect of malabsorption-induced VDD is inconsequential, given that healthy individuals with proper nutrition and intact intestinal absorption are still prone to VDD from lack of UVB exposure, the major vitamin D provider in the human body [30]. Other gastrointestinal-associated conditions, such as biliary diseases requiring cholecystectomy, have also been reported to be associated with lower 25(OH)D, yet its exact pathophysiology is unclear [32].

Kidneys play a significant role in activating vitamin D by converting its inactive form, 25(OH)D, to its active form, calcitriol [33]. In chronic renal impairment, renal 1α-hydroxylase expression is diminished by the effect of the hyperphosphaturic osteocyte-derived hormone (FGF-23), which increases to counterbalance the retention of phosphate in these patients [33]. Moreover, FGF-23 enhances 24-hydroxylase expression, which degrades calcitriol [33]. In addition, renal excretion of vitamin D-binding protein is observed in nephrotic syndrome, which also contributes to VDD [31,33,34]. Age and female gender are also correlated with VDD among those with chronic renal impairment [33,34].

Evidence proves that the metabolism and function of vitamin D are also altered by adiposity [3]. Observational studies have conveyed the increased risk of VDD among obese individuals, but with an uncertain direction of causality [3]. However, a bi-directional Mendelian randomization meta-analysis proves that the most likely clarification for this observation is the larger capacity of AT to store vitamin D in obese individuals, which results in lower circulating 25(OH)D levels [3]. The interrelation between obesity and vitamin D status was consistent and linear among different populations, all age groups, and both genders [3]. Nevertheless, it was shown that a larger BMI is a causative factor for VDD, but it offered no evidence for the fundamental role of vitamin D in inducing obesity [3].

Vitamin D is known to coordinate with magnesium to optimize each other’s functions; vitamin D can enhance intestinal magnesium absorption, and the latter is a cofactor in both hepatic and renal vitamin D activation reactions. Therefore, deficient magnesium states also cause VDD [35]. Primary hyperparathyroidism (PHPT), secondary to benign parathyroid adenomas, has also been largely associated with low serum 25(OH)D levels in several studies [36,37]. The degree of serum PTH elevation in these patients and the weight of adenoma were both inversely correlated with 25(OH)D levels, and both clinical and biochemical attributes of PHPT were intensified with the co-existing VDD [36,37]. Moreover, vitamin D status has been shown to improve without the need for supplementation when PHPT was treated [38].

Melanin is an extremely efficient UVB radiation absorbent. Therefore, dark-skinned people have an increased risk of VDD as high melanin in their skin absorbs the majority of UVB upon sun exposure, reducing dermal vitamin D synthesis as compared to those with white skin [25,30].

Throughout pregnancy and while breastfeeding, fetal nutritional demands, especially for bone formation, are met by consuming maternal vitamin D and calcium stores, predisposing to maternal VDD [39]. Drug-induced VDD should also be looked at; some drugs can lead to VDD both directly and indirectly, or by inducing degradation of the vitamin, such as some anticonvulsants, rifampicin, and glucocorticoids [25,40].

## 5. Cellular Senescence

### 5.1. Overview of Cellular Senescence

Cellular senescence came into sight over the past decade as an origin of dysfunctional cells and aging. It is defined as the stress-induced permanent termination of cell growth [41,42]. Telomere erosion, genomic damage, oxidative stress, genetic instability, and oncogenic activation are all cellular stressors that induce senescence at both the cellular and molecular levels [41,42,43]. Despite the well-known destructive effects of senescent cells in augmenting several age-associated conditions, such as atherosclerosis, cardiovascular diseases, renal insufficiency, neurodegeneration, glaucoma, and much more [42,43], senescence also plays a physiologically fundamental role in normal development, tumor suppression, tissue repair, and maintaining tissue homeostasis [41,42,43]. A thorough understanding of the senescence mechanism to avoid its downsides and gain its upsides is challenging.

### 5.2. The Theory of Aging and Cellular Senescence

It has been decades since “The Free Radical Theory of Aging” (FRTA) has been proposed. It stated that the aging of organisms arises from the accumulation of oxidative damage by reactive oxygen species (ROS), which are metabolites of molecular oxygen that are byproducts of various processes such as the electron transport chain (ETC) [44,45]. This theory was generated from various research showing the lifespan of an organism to shorten with ROS and lengthen with eliminating their oxidative damage [44,45]. Since then, the domination of this theory has taken place as the only explanation of aging, hence, cellular senescence [44,45].

However, emerging evidence has shown contradicting results. For instance, some studies have proven that while antioxidants may have protective effects, overexpression might sometimes induce a shorter lifespan, and their insufficient role in combating oxidative damage has also been documented [44,45]. Additionally, aging has been observed even in anaerobic states, where little ROS are generated, implying other causes of aging and senescence beyond oxidative damage [44]. Other suggested causes for aging include transcriptional and translational errors generating malfunctioning proteins, DNA damage, directing proteins to cells other than target organs, as well as metabolites’ damage. The accumulative effect of damage from all these factors, besides ROS, has been the preferable idea for the cause of aging [44]. It has also been suggested that the heterogeneity of molecules and biological processes also partakes in the occurring damage. For example, despite the high specificity of enzymes for their substrates, they also generate undesirable byproducts that may indirectly react with molecules other than their intended substrates, both of which are genetically determined [44].

### 5.3. Risk Factors of Cellular Senescence

**Figure 1:**
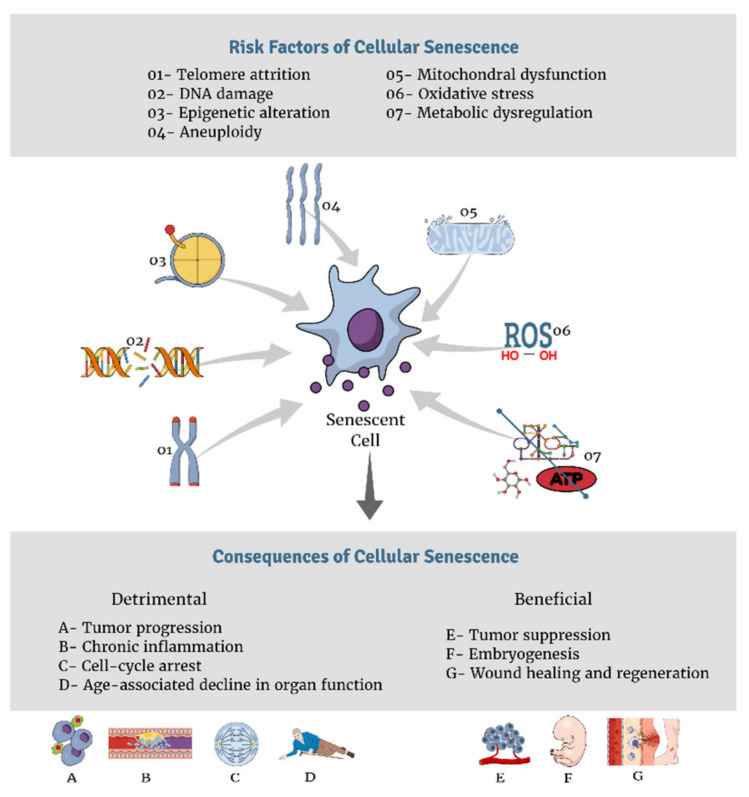
Risk factors and consequences of cellular senescence.

The above mentioned destructive effects do not lead to aging right away, it is the accumulative effect of damage that leads to cell growth arrest and the “senescence-associated secretory phenotype” (SASP), a hallmark of senescence through which cells transform fibroblasts into pro-inflammatory cytokines that harbors deleterious effects on the microenvironment of tissues [46,47]. This ultimately causes visible macroscopic outcomes at the organismal level, including tissue destruction and the generation of the physiologically aged phenotype [47]. Multiple factors disturb cellular generation, and in turn, enhance senescence. Figure 1 summarizes various risk factors and consequences of cellular senescence. The following is an analysis of a few of the major drivers of cellular senescence.

#### 5.3.1. Telomere Shortening

The capability of cells to proliferate is largely determined by chromosomal structure and function, both of which are preserved by telomeres [47,48]. Telomeres are repetitive DNA sequences at the end of every chromosome and are capped by a protective multi-protein complex called Shelterin [43,47,48]. Shelterin ensures that DNA damage responses are not activated, thereby preventing telomere crisis [43]. Telomeres can elongate by the action of telomerase, an enzyme that is suppressed in human somatic cells [49], therefore telomeres get uncapped and shorten with repetitive cell division [48]. Telomere length and the activity of telomerase are highly regulated in humans to maintain chromosomal stability [47]. When telomerase was expressed ectopically, the effects of telomere shortening were counterbalanced [48]. Telomere attrition and shortening consequently result in promoting DNA damage response, which ultimately leads to cell-cycle termination called replicative senescence [47,48].

#### 5.3.2. Genomic Damage

DNA damage, such as double-strand breaks, provokes DNA damage response (DDR) signals [47]. These signals activate various DDR-associated proteins, such as p53, which initiates senescence, particularly SASP [47,50]. Consequently, SASP initiates a cascade of pro-inflammatory cytokines that spreads to the neighboring and remote environment of damaged cells, promoting age-associated diseases [50]. Interleukins, chemokines, and growth factors are also implicated in the DDR/SASP response [50]. When genomic damage is accompanied by oxidative stress, the condition exacerbates [51].

#### 5.3.3. Metabolic Dysfunction

Impaired cellular metabolism provides a suitable environment for senescence and aging [43,47,48]. Various studies have observed the slower aging process with caloric restriction (CR) [43,52]. For instance, improved insulin sensitivity in metabolic regulation has been associated with a longer life span through CR [43,53]. Additionally, the mTORC pathway, a central regulator of metabolism, interconnects growth signals and nutrients to regulate various vital cellular processes, such as lipids and protein production and metabolism, and consequently, mTORC partakes in the process of the regulation of senescence [43].

#### 5.3.4. Mitochondrial Dysfunction and Oxidative stress

Mitochondrial dysfunction, with the resultant inadequate ATP supplies, leads to the inability of cells to self-replicate due to insufficient energy [47,54]. In addition, ROS generated by dysfunctional mitochondria has been known to largely contribute to aging and cellular senescence [54], mainly via telomere damage [47,55], and oncogenic activation [56]. Mitochondrial DNA (mtDNA) damage, and not only its functional dysregulation, can also promote aging and senescence [47,57]. In mice with dysfunctional mtDNA polymerase, defective proof-reading was observed, with multiple mtDNA mutations, and an end result of a premature aging process [57].

#### 5.3.5. Other Risk Factors of Senescence

Epigenetic alterations, without affecting DNA sequences, were also found to promote cellular senescence [58]. Both hypo and hyper-methylation of different chromosomal regions of various cell types have been reported to be associated with senescence in many studies [47]. Besides the previously mentioned factors, aneuploidy has also been known to induce cellular senescence [47], mainly by inhibiting the expression of the mitotic checkpoint protein (BubR1) [47]. BubR1 is a known contributor to age-related conditions, while its activation was shown to be protective against aneuploidy, with a prolongation of lifespan [59].

## 6. The Interrelation between Obesity, Vitamin D Deficiency, Cellular Senescence, and Obesity-Related Co-Morbidities

### 6.1. The Link between VDD and Obesity

The bi-directional relationship between VDD and obesity has been extensively explored in the literature [1,3,60]. Obesity is well-known to associate with a higher risk of VDD in all ages [1,3]. However, obesity does not seem to be induced by deficient vitamin D states [1,3], but this is not a consistent finding between all studies. Conversely, a systematic review of cohort studies looked at the causal relationship between the two conditions and indicated that VDD contributes to obesity occurrence [60]. Despite the debate regarding the causality between VDD and obesity, the role of VDD in promoting obesity-related consequences is out of the question, and calcitriol is known for its potential to drive down obesity-related damage [1]. In this section, we summarized some possible mechanisms to clarify the link between VDD and obesity.

#### 6.1.1. Vitamin D Uptake, Storage, and Metabolism in the Adipose Tissue

Since the majority of vitamin D in humans is stored in AT [1,61], it is believed that the large bulk of body fat pool in obese individuals sequesters vitamin D, hence reducing circulating 25(OH)D levels [1,3,62]. Serum 25(OH)D directly correlates with the total AT content of 25(OH)D [1]. Upon sun exposure, circulating 25(OH)D levels were shown to be slightly lower in obese individuals as compared to lean individuals, which could be explained by the fast uptake of vitamin D by AT, which in turn reduces the supplies of vitamin D metabolites needed for target tissues [1]. This also results in serum volumetric dilution that decreases 25(OH)D levels [1]. Moreover, AT expresses enzymes for both the formation and degradation of vitamin D [1,61,63]. Lower levels of both CYP2J2 and CYP27B, encoding for hydroxylases, are observed in obese individuals, which reduces their vitamin D activation [1,61]. It has also been hypothesized that vitamin D activation enzymes in AT explain the greater local use of vitamin D within AT, reducing its circulating levels [63]. AT also expresses VDR, which is necessary for calcitriol actions to be elicited, as well as CYP24A1, which degrades calcitriol; thus, AT is a major regulator of vitamin D metabolism [1]. This regulation also prevents toxicity from liberating unnecessary amounts of vitamin D and its metabolites into circulation [1]. Despite their lower serum 25(OH)D levels, obese people do not exhibit VDD adverse effects on bone, most likely due to the larger vitamin D stores they possess, which keep up with their needs [62].

#### 6.1.2. Alteration of Cutaneous Vitamin D Production in Obesity

Although obese individuals possess a larger surface area and increased vitamin D synthesis is expected [61], the opposite has been observed in some studies. A population-based study demonstrated an inverse relationship between sun exposure and BMI [63,64]. When sunbathing habits were assessed across different BMI groups, obese individuals were shown to avoid sun exposure, which in turn reduces their vitamin D syntheses [64]. Interestingly, the conversion of the cutaneous substrate of vitamin D (7-dehydrocholesterol) into pre-vitamin D by ultraviolet light was found to be maintained among obese individuals exercising outdoor, thus preserving their vitamin D synthesis capability [61].

#### 6.1.3. Surgically Induced Malabsorption

Bariatric surgeries, such as gastric bypass, cause a postoperative state of malabsorption of different nutrients, including vitamin D [61]. However, no evidence proves that dietary vitamin D absorption is lowered by obesity. Fortunately, the magnitude of disturbance of vitamin D–calcium homeostasis among those with malabsorption is trivial; since vitamin D is fat-soluble, high-fat diets have been shown to increase calcium absorption [61].

#### 6.1.4. Effects of Weight Loss on Vitamin D Concentration

Considerable evidence proves that weight loss improves serum 25(OH)D levels, with numerous studies reporting their positive correlation [1,61,65]. However, 25(OH)D elevation is not dramatic, which might be secondary to the inactivation of vitamin D by AT, or increased sequestration of vitamin D metabolites by target tissues after their release into the bloodstream with weight loss, especially when abdominal fat is lost [1,66]. A two-year clinical trial on 383 obese or overweight women taking part in a weight-loss program showed that the mean increase in calcidiol level was 1.9 ng/mL among those who did not lose weight, while the level continuously increased and reached 5.0 ng/mL for participants losing more than 10% of their baseline weight (*p* = 0.014), indicating a positive association between weight loss and calcidiol levels [65].

#### 6.1.5. Effects of Vitamin D Supplementation on Obesity-Related Disorders

There is insufficient evidence regarding the utilization of vitamin D as a preventive measure against obesity-related consequences. In various studies and clinical trials, calcitriol was shown to activate anti-inflammatory and inhibit pro-inflammatory cytokines in obese individuals with low-grade inflammation [67,68]. In addition, adiponectin [69] and insulin-like growth factor-1 (IGF-1) [70] were both seen to be upregulated by calcitriol, both of which are protective against inflammation and metabolic syndrome, respectively [1]. Moreover, those with obesity-induced IR and hyperglycemia could gain some benefits from calcitriol via its ability to enhance healthy pancreatic beta cells to secrete insulin [71], and protect these cells from hyperglycemic effects [72]. Calcitriol also positively impacts obese individuals with hypertriglyceridemia by reducing triglycerides production in the liver and inhibiting elongase enzyme that is responsible for lengthening fatty acids to promote long-chain formation, which exacerbates the condition [1,73]. Last but not least, renin–angiotensin system inhibition was achieved by calcitriol, which helps hypertensive obese patients preserve their renal function [74].

**Figure 2:**
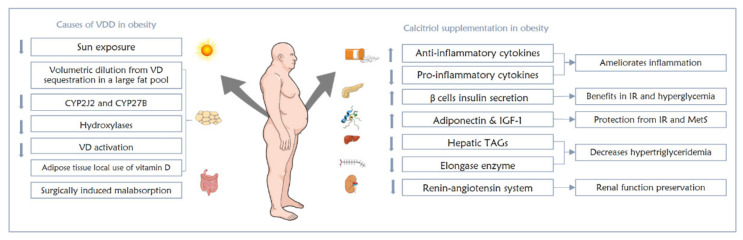
The interrelation between VDD and obesity and the effects of calcitriol supplementation on obese individuals.

In spite of the positive proven role of calcitriol in obesity comorbidities, drastic effects should not be expected [1,63]. A meta-analysis proposing that the response of obese individuals to vitamin D supplementation is reduced showed that obese individuals preserve lower amounts of the supplemented vitamin D as compared to euphoric people, partly due to the large AT sequestration of administered vitamin D, and no additional benefits were found from higher doses of vitamin D supplements [63]. The link between VDD and obesity is summarized in Figure 2.

### 6.2. The Link between Cellular Senescence and Obesity

Obesity, IR, and T2DM provide a favorable environment that promotes cellular senescence and accelerated age-related conditions [75]. The role of senescent cells in obese AT as a promotor of IR has been investigated in various studies [75]. The higher AT mass associated with obesity is mainly linked to the enlargement of adipocytes that store energy [75]. In mice with excessive caloric intake, oxidative stress has been increased in AT which enhanced senescence-like changes, such as the overexpression of p53 and increased activity of senescence-associated β-galactosidase (SA-β-Gal), both of which are markers of cellular senescence, besides the elevation of pro-inflammatory cytokines [76]. Senescence marker protein 30 (SMP30) has an anti-apoptotic protective role, mainly by regulating Ca^2+^ homeostasis [77], and it was also shown to be largely reduced in obese rats, and normalized with vitamin D supplementation [78]. AT of diabetics also showed elevated markers of cellular senescence [75]. P53, a promoter of IR, has been upregulated in the AT of obese mice and associated with glucose intolerance [75]. Pifithrin-α, a p53 deactivator, was shown to slow down the cellular senescence of adipocytes, limit metabolic disturbances [79], and improve IR [75].

Furthermore, when adipocytes were subject to oxidative stress to induce senescence, ROS formation, genomic damage, telomere shortening, overexpression of p53, p21, and inflammatory cytokines were all increased [80]. Adiponectin was also reduced in these cells leading to dysfunctional glucose metabolism with reduced cellular glucose uptake, which predisposes to T2DM [80]. Moreover, the differentiation of adipocytes has been shown to be hindered by senescence [81]. Stromal cells originating from senescent adipocytes have an abnormal expression of genes that play a role in their differentiation, as well as lower expression of key regulators of adipogenesis when subjected to certain adipogenic hormones [81].

The association between obesity-related high lipopolysaccharides (LPS) and the enhancement of cellular senescence has been looked at [82]. When LPS was administered into adipocytes progenitors in mice, stromal-vascular cells that were isolated from these tissues showed induced irreversible cellular senescence in the form of under-expression of mRNA of adipocyte marker genes, p53 and SA-β-gal activity activation, ROS elevation, and the development of the SASP phenotype [82]. Additionally, activin A released by human senescent adipocyte progenitor cells (APCs) can inhibit adipogenesis in young mature cells, which was observed to be reversible after eliminating senescent cells in mice [83]. Thus, via various mechanisms, senescent adipocytes may promote obesity, T2DM, IR, and their associated age-related dysfunction.

### 6.3. The Link between VDD and Cellular Senescence

The evidence shows that vitamin D synthesis is reduced with aging, the outcome of senescence. Elderly people have reported around 70% lower dermal vitamin D production as compared to younger ones [84]. 7-dehydrocholesterol, the precursor for dermal vitamin D synthesis, is largely reduced with aging due to age-associated dermal structural alterations, such as shrinkage and reduced elasticity [84]. Moreover, the age-related deterioration in renal function impairs calcitriol production [85].

It has been hypothesized that calcitriol bears an antiaging effect through decreasing ROS and genomic damage, which enhances the proliferative ability of cells, inhibits SASP, and upregulates the cytoprotective nuclear factor (Nrf2) [86], and in turn, promotes the clearance of ROS and damaged proteins [87]. Various studies have proven vitamin D replacement to overcome oxidative damage induced by ROS [86,88]. The average lifespan of mice treated with calcitriol was nearly five times longer as compared to those who were calcitriol-deficient and had multiple aging phenotypes in various organs [86]. Additionally, exogenous calcitriol inhibited SASP, ROS, and genomic damage in the calcitriol-treated group, and promoted cellular proliferation [86]. Besides, this aging-protective function of vitamin D is augmented by its ability to increase the expression of the protein Klotho which has anti-aging functions [89]. Mutations in the Klotho gene have been shown to induce premature aging syndrome in mice, as reported by KurO-O [90]. Accordingly, the vitamin D–Klotho–Nrf2 signaling network is considered a major aging regulator [91]. At optimal vitamin D levels, vitamin D, Klotho protein, and Nrf2 collectively act to normalize and slow down the aging rate, while at deficient vitamin D states, aging processes are accelerated [91].

Current evidence postulates that vitamin D partakes in preserving genomic stability and telomere length, which are direct determinants of cellular senescence [92]. The role of Vitamin D in regulating telomerase activity has been widely researched [93,94,95]. Vitamin D was found to exhibit anti-inflammatory and anti-proliferative properties that aid in maintaining telomeres length [92]. In vitamin D-deficient patients, telomeres were shorter than in those who received vitamin D supplements [95]. The positive correlation between telomere length and vitamin D is concentration-dependent, as supported by a study on women conducted by Liu and colleagues [94].

### 6.4. Subclinical Atherosclerosis

Atherosclerosis (ATH) is a lipid-induced, chronic, progressive disorder, identified by a low-grade inflammatory state and the build-up of lipids and fibrous tissue in arterial walls [96,97]. It initiates in sites with endothelial dysfunction, and it is usually an asymptomatic condition at its early stages [96]. Breaking the integrity of the athero-protective endothelial cells in arterial walls, such as with hypertension or turbulent blood flow, predispose it to atherosclerosis [96,97]. Obesity promotes the build-up of fat in arterial walls, and ultimately the accompanying inflammation [96,97]. Other factors that partake in atherosclerosis development include genetic orientation, smoking [98], hypertension, sedentary lifestyle, and obesity. The scope of causative factors of atherosclerosis is relatively broad [99].

#### 6.4.1. Obesity and Subclinical Atherosclerosis

Obesity vascular alteration in obesity is subject to various factors [100]. Early subclinical atherosclerosis is promoted by cardiometabolic risk factors [5]. Various studies have determined that visceral AT is a metabolically active organ, and its role in the cardiometabolic risk involves its ability to secrete hormones, adipocytokines, inflammatory mediators, growth factors, and fibrinolysis markers [101]. The association between subclinical atherosclerosis and two adipokines of opposing effects, adiponectin, and leptin, was examined by measuring coronary artery calcification (CAC) as a determinant of subclinical atherosclerosis [102]. The anti-inflammatory and athero-protective adiponectin, which also improves insulin sensitivity, was found to be low in obesity and coronary artery disease (CAD), while leptin, an immunomodulatory and IR promoter that is elevated in obesity, was strongly correlated with obesity and independently with CAC [102]. Adiponectin is believed to provide athero-protection by inactivating the NF-κB inflammatory pathway in vascular cells [103], while leptin’s receptors are found to be expressed in atherosclerotic plaques [102,104]. Interestingly, subclinical atherosclerosis was found to be associated with adiposity even among those with MHO [105] and those with normal-weight obesity (NWO), which reflects those with normal BMI but increased body fat percentage that makes them vulnerable to cardiometabolic risk [106].

#### 6.4.2. VDD and Subclinical Atherosclerosis

The association between serum VDD and cardiovascular comorbidities has been confirmed in various studies [107,108,109,110,111]. Peripheral vascular diseases, cardiac ischemia, hypertension, and cardiac-associated mortality in general, are all highly prevalent among those with VDD [110]. Moreover, prospective studies have proven that VDD positively correlates with CAC and endothelial dysfunction [108,110], drives the growth and expansion of atherosclerotic plaques, stimulates vascular and systemic inflammation [107,109,111], and increases coronary artery calcium scores and vascular stiffness, which are all seen in subclinical atherosclerosis [108,109,112]. Studies have also highlighted the athero-protective effects of treatment with vitamin D [107,111].

The interrelation between subclinical atherosclerosis and VDD can be clarified by various mechanisms. Cardiomyocytes, endothelial cells, macrophages, lymphocytes, as well as vascular smooth muscle cells (VSMCs), all express receptors for vitamin D [109,113]. VSMCs produce prostacyclin under the influence of 25(OH)D which consequently plays a role in the protection against thrombosis, VSMCs proliferation, and cell adhesion [109,113]. Additionally, VDD promotes pancreatic β-cell dysfunction leading to IR and T2DM [114], as well as increasing the risk for hypertension by activating the renin-angiotensin system [74], and both are risk factors for subclinical atherosclerosis. Moreover, calcitriol suppresses cholesterol entry into macrophages, hence stopping foam cell formation in atherosclerosis, a lost benefit among those with VDD [115]. Given the crucial role of inflammation during the whole process of atherosclerosis development, from commencement up to full-blown thrombosis, the reduced anti-inflammatory effect of vitamin D and its metabolites when deficient can also explain the impact VDD has on atherosclerosis progression [116,117].

#### 6.4.3. Cellular Senescence and Subclinical Atherosclerosis

Vascular aging is not a sudden event. Instead, it happens in an ongoing manner with cellular, biochemical, and enzymatic involvement. Epigenetic and molecular changes are also crucial elements in its development [118]. Arterial aging largely determines the functions of different organs [118]. Vascular aging is characterized by arterial stiffness and hypertension secondary to low elastin and excessive collagen within arterial walls [118]. In view of that, early vascular aging (EVA) has emerged with accompanying cardiovascular risk and other age-related conditions [118]. EVA also appears to benefit clinical practice for those with high cardiovascular risk, such as strong genetic predisposition to premature cardiac events where early detection of the condition is possible [118]. Increased arterial stiffness, endothelial dysfunction, and vasodilation are the main aspects of this process [118]. It has been suggested that opposing this phenomenon can be achieved with an aggressive approach for controlling the risk factors of atherosclerosis [118].

However, strong evidence regarding its efficacy is lacking [119,120]. It is reported that shortened telomeres as an early sign and a characteristic feature of cellular senescence is a prognostic biomarker for the early identification of subjects with high cardiovascular risk [121]. The association between subclinical atherosclerosis and cellular senescence could be explained by generalized telomere shortening and dysfunction [121]. Telomere dysfunction induces metabolic and mitochondrial compromise [122], and the increased burden of oxidative stress can accelerate telomere shortening events [123]. Furthermore, telomere shortening is associated with increased serum levels of oxidized LDL (ox-LDL), which correlates with subclinical atherosclerosis [121,124].

### 6.5. Non-Alcoholic Fatty Liver Disease

The term NAFLD encompasses a range of hepatic disorders that occur with concomitant cardiometabolic disturbances, such as obesity and IR [125], and it reflects the hepatic manifestation of metabolic syndrome [126]. The hallmark of NAFLD is excessive fat accumulation within the liver (>5%) without considerable alcohol intake or any secondary causes of hepatic steatosis [125]. It can be grouped into simple steatosis (SS) and non-alcoholic steatohepatitis (NASH), which have the potential of progressing to liver cirrhosis as well as hepatocellular carcinoma (HCC) [6,125]. Various genetic and environmental factors enhance NAFLD evolvement, some of which include a fat-rich diet and sedentary lifestyle with their accompanying obesity and IR, epigenetic modifications, lipotoxicity, and oxidative stress [6].

#### 6.5.1. The Association between NAFLD and Obesity

Since obesity has been a significant contributor to the progression and severity of NAFLD [6], the link between both will be explained. Obesity partakes in the development of the NAFLD continuum, from SS to NASH. In obese individuals with adipocytes fully saturated with energy storage, hepatocytes can play the role of adipocytes, hence the shifting of excess lipids into hepatocytes for storage as TAGs, generating SS [6]. Furthermore, when TAGs hydrolysis is promoted, and the uptake of the resultant FFAs are diminished in adipocytes, their overload in the circulation enhances their ectopic accumulation in the liver and other sites. Subsequently, TAGs metabolites will cause lipotoxicity [6,125,127]. The well-known mechanisms linking lipotoxicity to NAFLD are dysfunctional mitochondria, activation of inflammatory pathways, and lipoapoptosis, which arises due to hepatocytes’ inability to eliminate FFAs [6,125,127]. Afterwards, as SS develops, and if obesity was left untreated at this stage, innate immune cells, such as Kupffer and dendritic cells, gain access to hepatocytes and inflammation begins, which hallmarks the progression to NASH [6]. At this point, the liver is infiltrated with macrophages, neutrophils, T-lymphocytes, and cytokines, which exacerbate the condition. As the inflammatory phase is prolonged, fibrotic changes start to occur [6].

Under normal conditions, the liver tissue attempts to regenerate damaged hepatocytes, which is successfully achieved by the coordinated mechanisms between wound healing cells and immune cells: endothelial cells, progenitor cells, and myofibroblasts, while in long-term obesity, hepatocytes fail to sustain these mechanisms, thus fibrosis develops with the outcome of cirrhosis or HCC [6]. Another mechanism by which obesity distresses the liver is by adipocytes-derived adipokines [6,125], such as adiponectin and leptin, which lose their homeostasis in obesity [6]. When adipocytes expand in obesity, they become dysfunctional and inflamed [125]. The released adipokines tend to induce steatogenesis in the liver and inflammation. As inflammatory cells, mainly neutrophils and lymphocytes, further infiltrate adipocytes as they expand, interleukins (IL) 1 and 6 as well as TNF-α crosstalk with adipokines, aggravating steatosis [6,125].

Despite the clear role of adipokines in promoting fatty liver, they are not always foreseeable to promote steatogenesis, as adipokines may have a two-sided action [6]. It has been observed that in mice with different stages of NAFLD, leptin enhances hepatic inflammation and fibrosis only as the condition progresses. In contrast, at the early stages of steatosis, leptin had an anti-steatotic effect [6]. Furthermore, in humans, low leptin concentrations were observed in healthy individuals, and its gradual elevation was seen as NAFLD progressed to SS, then to NASH [6]. Contrary to leptin, low adiponectin levels have been noted with visceral fat mass enlargement. This is because adiponectin exhibits anti-inflammatory and anti-steatotic properties by inhibiting pro-inflammatory cytokines and promoting anti-inflammatory ones, such as IL-10, which lessens oxidative stress and hepatic fibrogenesis [6]. Adiponectin levels have been observed to gradually decline in humans with NAFLD as the disease progresses, except when cirrhosis develops and hepatocytes fail to eliminate excess adiponectin [6]. Conclusively, higher adiponectin levels have been associated with a poorer prognosis in NAFLD patients [6].

#### 6.5.2. The Association between NAFLD and VDD

Because of the known associations between obesity and NAFLD and between obesity and VVD, expanding evidence links the association between VDD and NAFLD, which is explained in part in this review. The histological features of NAFLD such as the degree of steatosis, inflammatory infiltration, necrosis, and fibrosis have been closely linked with VDD with disregard to other factors, such as age, sex, Homeostatic Model Assessment (HOMA)-IR score, and co-existing metabolic disturbances [128]. Roth et al. have also shown that VDD contributed to NAFLD progression via activating toll-like receptors (TLR), promoting IR, and overexpressing genes responsible for oxidative stress and inflammation [129]. Additionally, Liangpunsakul et al. found that an unexplained hepatic impairment was linked to low vitamin D levels despite controlling other factors, such as TAGs, IR, and metabolic syndrome [130]. Cordeiro et al. demonstrated that the lowest vitamin D levels were observed in the serum of those with steatohepatitis on hepatic biopsies, especially with late stages of NAFLD [131].

The exact way by which VDD contributes to NAFLD is not very clear. However, a part of its contribution was explained by some mechanisms. Vitamin D performs its functions by acting upon its hepatic receptor, vitamin D receptor (VDR), and defects in vitamin D-VDR axis signaling might explain why the expression of hepatic VDR was shown to be inversely associated with the extent of NAFLD, which further justifies the interrelation between VDD and NAFLD [131]. It is also believed that VDD can intensify NAFLD via inflammatory-mediated and immunomodulatory processes and somewhat by the inhibition of its anti-inflammatory functions [131]. Nevertheless, it cannot be ascertained that NAFLD does not also worsen vitamin D status; since liver-hydroxylation is an important step in vitamin D synthesis, hepatic impairment can also clarify why vitamin D stores are depleted in most liver disorders [131].

Vitamin D replacement therapy has been linked with the improvement of NAFLD in various studies. The effect of artificial sunlight-induced vitamin D elevation on NASH among rats have been explored, and the reduction in hepatic inflammation and fibrotic process upon the increase in vitamin D was observed [132]. In addition to the improvement in IR, the elevation of adiponectin and the decrease in markers of hepatic stellate cell activation that are essential for the formation of myofibroblasts, such as hepatic transforming growth factor (TGF-β) and α-smooth muscle actin (α-SMA), were seen [132]. Hepatic histological improvement with vitamin D3 replacement was also noted [132]. Additionally, vitamin D-treated rats showed overexpression of senescence marker protein 30 (SMP30), which is inversely related to NAFLD progression [126].

Genome-wide studies have emphasized the role of vitamin D pathway genes in many processes that are strongly linked to NAFLD, such as immunological alteration, cellular differentiation, and inflammatory processes [133,134]. Various genetic studies have revealed frequent single nucleotide polymorphisms (SNPs) in vitamin D pathway genes and highlighted their association with low serum vitamin D levels both in healthy subjects and those with hepatitis, such as the genes for CYP2R1 and CYP27B1 [133], while mutations in the CYP24A1 gene, responsible to vitamin D degradation [135], causes calcitriol elevation [136]. A case-control study that investigated the effect of vitamin D gene SNPs has shown that alleles CYP24A1 rs2296241-A, rs2248359-T, and CYP27B1 rs4646536-T were all considered a risk factor for NAFLD when combined with other clinical factors [134]. Additionally, these alleles were found to co-exist in some NAFLD patients [134]. Another study that determined SNPs of the VDR gene in NAFLD-proven subjects with liver biopsy has shown VDR rs1544410 genotype CC to be independently correlated with advanced liver fibrosis [133]. Other studies have suggested the role of VDR, VDBP gene polymorphisms in the progression of liver diseases such as inflammation and liver fibrosis [133].

#### 6.5.3. The Association between NAFLD and Cellular Senescence

In recent times, the interest of researchers has grown to investigate the relationship between cellular senescence and NAFLD progression. The metabolic disturbances and hepatic inflammatory processes in NAFLD might arise from or participate in cellular senescence, denoting that either condition may provoke the other [137]. Obesity-prone rats on a fat-rich diet with severe NAFLD expressed higher mRNA of p16 and p21 senescence-associated pathways, lower p53 (a cell cycle regulator), and increased histones H3 and H4 acetylation [138]. Another study also revealed that mice with fatty liver expressed various senescence markers in the hepatocytes, including γH2AX that is related to senescence-induced damage foci, increased senescence-related distention of satellites, and larger nuclear areas, and further, that genetically eliminated senescent cells expressing p16 showed an improvement in steatosis [139].

It has been discovered that senescence-linked proteins also relate to the course of NAFLD. The antioxidant and anti-apoptotic SMP30 is involved in NAFLD progression [137]. SMP30 knockout mice have been shown to exhibit hepatic steatosis and increased hepatic oxidative stress compared to wild-type mice. This mechanism may be related to lower apolipoprotein B in the liver and dysfunctional lipid metabolism [137].

Additionally, when NASH was induced by methionine and choline-free diet into p53 deficient mice, the course of NAFLD was slowed down, oxidative stress dropped, and apoptosis was decreased [137]. In addition to the p53 effects as an oxidative stress reducer, it was also of interest to explore what enhances oxidative stress in the liver, and TGF-β was proven to be a ROS enhancer [137]. Collectively, these findings point out the overexpression of senescence-related proteins in cases of hepatic steatosis. Given that cellular senescence is aging cells, it is anticipated that senescence is promoted by aging itself, contributing to the course of NAFLD via multiple mechanisms. Age-associated mitochondrial dysfunction and enhanced oxidative stress generated steatosis in mice on a fatty diet. Furthermore, the aging-induced upregulation of the cyclin-dependent kinase-4 (cdk4) phosphorylates CCAAT enhancer-binding protein (C/EBP α) and formation of C/EBPa-p300 complexes causing steatosis, and CDK-4 inhibition, was shown to lower steatosis [137,140].

The link between NAFLD and senescence has also been widely considered in humans. Hepatic histology confirmed that telomere-associated damage foci and p21 are associated with NAFLD severity [139]. P53 levels were also higher in the livers of humans with NAFLD as compared to healthy controls [137]. Shortening of telomeres and lowering of the nuclear area were both histologically observed in livers of those with NAFLD, as well as the expression of γH2AX, which implies DNA damage along with the elevation of p21 that accompanies cell cycle arrest [141]. Genetic variants in the gene encoding p21 protein (CDKN1A) may also participate in NAFLD progression, but once fibrosis develops, no further progress has been observed with some of its SNPs [142]. Other features of DNA damage are also reflective of cellular senescence and were discovered to be upregulated in NAFLD, such as cellular micronuclei, nucleoplasmic bridges, and nuclear buds [137].

Furthermore, altered DNA methylation patterns have been associated with the evolution of the NAFLD course and discriminated between patients in different NAFLD stages [137]. An example is the PPARG gene, which regulates the storage of lipids and glucose metabolism, which was found to be hypermethylated in the hepatic tissues of NAFLD individuals [143]. Despite all recognized mechanisms that relate senescence to NAFLD, additional research is needed to elucidate their exact interrelation.

#### 6.5.4. The Association between NAFLD and Subclinical Atherosclerosis

There is a substantial current build-up in the literature clarifying the connection between NAFLD and subclinical atherosclerosis. In the absence of any existing cardiometabolic disorders, NAFLD has been associated with dysfunctional endothelium, vulnerable high-risk atherosclerotic plaques, CAC, and carotid artery inflammation, which are all markers of subclinical atherosclerosis [144,145]. Subclinical atherosclerosis is prevalent among patients with NAFLD due to mutual risk factors between the two conditions, such as obesity, IR, hyperlipidemia, hypertension, renal impairment, and hyperuricemia. Additionally, NAFLD patients have elevated inflammatory markers such IL-6, TNF-α, and high sensitivity C-reactive protein (Hs-CRP), as well as the pro-coagulant fibrinogen, which are all key elements in the pathophysiology of atherosclerosis [145,146]. The athero-protective adiponectin has also been observed to be low among those with NAFLD [145].

At present, NAFLD/NASH and subclinical atherosclerosis are believed to be two sides of the same coin, which is the shared disease with mutual origins, risk factors, and inflammatory elements. However, NAFLD is not guiltless when it comes to atherosclerosis development. It retains pro-atherogenic properties that accelerate atherosclerosis, such as its crosstalk with IR, disturbed lipoproteins metabolism, long-term inflammatory state, ROS formation, and the drop in athero-protective adiponectin levels [147]. Conclusively, precise biological processes linking NAFLD to subclinical atherosclerosis remain uncertain.

## 7. Conclusions

In conclusion, subclinical atherosclerosis and NAFLD are common disorders that are associated with obesity. VDD is a prominent event usually associated with obesity, mainly as an effect. Both obesity and VDD play a role in the pathogenesis of subclinical atherosclerosis and NAFLD by a variety of pathogenic mechanisms, and it is proved that accelerated cellular senescence could be one of the important mechanisms contributing to both diseases.

## References

[1] Hyppönen E., Boucher B.J. (2018). Adiposity, vitamin D requirements, and clinical implications for obesity-related metabolic abnormalities. Nutr. Rev..

[2] Ogrodnik M., Zhu Y., Langhi L.G., Tchkonia T., Krüger P., Fielder E., Victorelli S., Ruswhandi R.A., Giorgadze N., Pirtskhalava T. (2019). Obesity-Induced Cellular Senescence Drives Anxiety and Impairs Neurogenesis. Cell Metab..

[3] Vimaleswaran K.S., Berry D.J., Luben R., Tikkanen E., Pilz S., Hiraki L.T., Cooper J.D., Dastani Z., Elliott P., Houston D. (2013). Causal Relationship between Obesity and Vitamin D Status: Bi-Directional Mendelian Randomization Analysis of Multiple Cohorts. PLoS Med..

[4] Vranić L., Mikolašević I., Milić S. (2019). Vitamin D Deficiency: Consequence or Cause of Obesity?. Medicina.

[5] Laing S.T., Smulevitz B., Vatcheva K.P., Rahbar M.H., Reininger B., McPherson D.D., McCormick J.B., Fisher-Hoch S.P. (2015). Subclinical Atherosclerosis and Obesity Phenotypes among Mexican Americans. J. Am. Heart Assoc..

[6] Polyzos S.A., Kountouras J., Mantzoros C.S. (2019). Obesity and nonalcoholic fatty liver disease: From pathophysiology to therapeutics. Metabolism.

[7] World Health Organization (2020). Overweight and Obesity.

[8] Ross R., Neeland I.J., Yamashita S., Shai I., Seidell J., Magni P., Santos R.D., Arsenault B., Cuevas A., Hu F.B. (2020). Waist circumference as a vital sign in clinical practice: A Consensus Statement from the IAS and ICCR Working Group on Visceral Obesity. Nat. Rev. Endocrinol..

[9] Stefan N., Häring H.-U., Hu F.B., Schulze M.B. (2013). Metabolically healthy obesity: Epidemiology, mechanisms, and clinical implications. Lancet Diabetes Endocrinol..

[10] Phillips C.M. (2016). Metabolically healthy obesity across the life course: Epidemiology, determinants, and implications. Ann. N. Y. Acad. Sci..

[11] Pischon T., Boeing H., Hoffmann K., Bergmann M., Schulze M.B., Overvad K., Van Der Schouw Y., Spencer E., Moons K., Tjønneland A. (2008). General and Abdominal Adiposity and Risk of Death in Europe. N. Engl. J. Med..

[12] Wang Y.C., McPherson K., Marsh T., Gortmaker S.L., Brown M. (2011). Health and economic burden of the projected obesity trends in the USA and the UK. Lancet.

[13] Kansra A.R., Lakkunarajah S., Jay M.S. (2021). Childhood and Adolescent Obesity: A Review. Front. Pediatr..

[14] Nakamura K., Fuster J.J., Walsh K. (2014). Adipokines: A link between obesity and cardiovascular disease. J. Cardiol..

[15] Fuster J.J., Ouchi N., Gokce N., Walsh K. (2016). Obesity-Induced Changes in Adipose Tissue Microenvironment and Their Impact on Cardiovascular Disease. Circ. Res..

[16] Goossens G.H. (2017). The Metabolic Phenotype in Obesity: Fat Mass, Body Fat Distribution, and Adipose Tissue Function. Obes. Facts.

[17] Del Valle H.B., Yaktine A.L., Taylor C.L., Ross A.C. (2011). Dietary Reference Intakes for Calcium and Vitamin D.

[18] Christakos S., Dhawan P., Verstuyf A., Verlinden L., Carmeliet G. (2016). Vitamin D: Metabolism, Molecular Mechanism of Action, and Pleiotropic Effects. Physiol. Rev..

[19] Bikle D.D., Schwartz J. (2019). Vitamin D Binding Protein, Total and Free Vitamin D Levels in Different Physiological and Pathophysiological Conditions. Front. Endocrinol..

[20] Bouillon R., Schuit F., Antonio L., Rastinejad F. (2020). Vitamin D Binding Protein: A Historic Overview. Front. Endocrinol..

[21] Holick M.F., Binkley N.C., Bischoff-Ferrari H.A., Gordon C.M., Hanley D.A., Heaney R.P., Murad M.H., Weaver C.M. (2011). Evaluation, Treatment, and Prevention of Vitamin D Deficiency: An Endocrine Society Clinical Practice Guideline. J. Clin. Endocrinol. Metab..

[22] De Azevedo F.R., Caramelli B. (2013). Hypovitaminosis D and Obesity—Coincidence or Consequence?. Eur. Endocrinol..

[23] Amrein K., Scherkl M., Hoffmann M., Neuwersch-Sommeregger S., Köstenberger M., Berisha A.T., Martucci G., Pilz S., Malle O. (2020). Vitamin D deficiency 2.0: An update on the current status worldwide. Eur. J. Clin. Nutr..

[24] Al-Daghri N.M., Hussain S.D., Ansari M.G., Khattak M.N., Aljohani N., Al-Saleh Y., Al-Harbi M.Y., Sabico S., Alokail M.S. (2021). Decreasing prevalence of vitamin D deficiency in the central region of Saudi Arabia (2008–2017). J. Steroid Biochem. Mol. Biol..

[25] Holick M.F., Chen T.C. (2008). Vitamin D deficiency: A worldwide problem with health consequences. Am. J. Clin. Nutr..

[26] Littlejohns T., Soni M., Annweiler C., Chaves P., Fried L., Kestenbaum B., Lang I., Langa K., Lopez O., Kos K. (2013). P4–394: Vitamin D and incident Alzheimer’s disease in the Cardiovascular Health Cognition Study. Alzheimer’s Dement..

[27] Schmid A., Walther B. (2013). Natural Vitamin D Content in Animal Products. Adv. Nutr..

[28] Chan J., Jaceldo-Siegl K., E Fraser G. (2009). Serum 25-hydroxyvitamin D status of vegetarians, partial vegetarians, and nonvegetarians: The Adventist Health Study-2. Am. J. Clin. Nutr..

[29] Weikert C., Trefflich I., Menzel J., Obeid R., Longree A., Dierkes J., Meyer K., Herter-Aeberli I., Mai K., Stangl G.I. (2020). Vitamin and Mineral Status in a Vegan Diet. Dtsch. Aerzteblatt Online.

[30] Margulies S.L., Kurian D., Elliott M.S., Han Z. (2015). Vitamin D deficiency in patients with intestinal malabsorption syndromes—Think in and outside the gut. J. Dig. Dis..

[31] Kennel K.A., Drake M.T., Hurley D.L. (2010). Vitamin D Deficiency in Adults: When to Test and How to Treat. Mayo Clin. Proc..

[32] Ekiz T., Yegen S.F., Katar M.K., Genç O., Genç S. (2018). 25-Hydroxyvitamin D levels and bone mineral density evaluation in patients with cholecystectomy: A case-control study. Arch. Osteoporos..

[33] Jean G., Souberbielle J.C., Chazot C. (2017). Vitamin D in Chronic Kidney Disease and Dialysis Patients. Nutrients.

[34] Caravaca-Fontán F., Gonzales-Candia B., Luna E., Caravaca F. (2016). Relative importance of the determinants of serum levels of 25-hydroxy vitamin D in patients with chronic kidney disease. Nefrología.

[35] Uwitonze A.M., Razzaque M.S. (2018). Role of Magnesium in Vitamin D Activation and Function. J. Am. Osteopat. Assoc..

[36] Silverberg S.J. (2007). Vitamin D Deficiency and Primary Hyperparathyroidism. J. Bone Miner. Res..

[37] Walker M., Cong E., Lee J.A., Kepley A., Zhang C., McMahon D.J., Silverberg S.J. (2015). Vitamin D in Primary Hyperparathyroidism: Effects on Clinical, Biochemical, and Densitometric Presentation. J. Clin. Endocrinol. Metab..

[38] Rodrigo A., Jorge S.-G., Eduardo M.-P., Caba M.D., Cordera F., Fernando C., Manuel M., Efraín C.-G., Fernando N.-C.L. (2018). Does Vitamin D Deficiency Cause Primary Hyperparathyroidism?. Int. J. Surg. Res. Pract..

[39] Mulligan M.L., Felton S.K., Riek A.E., Bernal-Mizrachi C. (2010). Implications of vitamin D deficiency in pregnancy and lactation. Am. J. Obstet. Gynecol..

[40] Gröber U., Kisters K. (2012). Influence of drugs on vitamin D and calcium metabolism. Derm.-Endocrinol..

[41] Rodier F., Campisi J. (2011). Four faces of cellular senescence. J. Cell Biol..

[42] Regulski M.J. (2017). Cellular Senescence: What, Why, and How. Wounds Compend. Clin. Res. Pract..

[43] McHugh D., Gil J. (2018). Senescence and aging: Causes, consequences, and therapeutic avenues. J. Cell Biol..

[44] Gladyshev V.N. (2014). The Free Radical Theory of Aging Is Dead. Long Live the Damage Theory!. Antioxid. Redox Signal..

[45] Ziegler D., Wiley C.D., Velarde M.C. (2015). Mitochondrial effectors of cellular senescence: Beyond the free radical theory of aging. Aging Cell.

[46] Coppé J.-P., Desprez P.-Y., Krtolica A., Campisi J. (2010). The Senescence-Associated Secretory Phenotype: The Dark Side of Tumor Suppression. Annu. Rev. Pathol. Mech. Dis..

[47] Ebhatia-Dey N., Kanherkar R.R., Stair S.E., Makarev E.O., Csoka A.B. (2016). Cellular Senescence as the Causal Nexus of Aging. Front. Genet..

[48] Victorelli S., Passos J. (2017). Telomeres and Cell Senescence—Size Matters Not. EBioMedicine.

[49] Collins K., Mitchell J.R. (2002). Telomerase in the human organism. Oncogene.

[50] Olivieri F., Albertini M.C., Orciani M., Ceka A., Cricca M., Procopio A.D., Bonafè M. (2015). DNA damage response (DDR) and senescence: Shuttled inflamma-miRNAs on the stage of inflamm-aging. Oncotarget.

[51] Davalli P., Mitic T., Caporali A., Lauriola A., D’Arca D. (2016). ROS, Cell Senescence, and Novel Molecular Mechanisms in Aging and Age-Related Diseases. Oxidative Med. Cell. Longev..

[52] Mitchell S.J., Madrigal-Matute J., Scheibye-Knudsen M., Fang E.F., Aon M., González-Reyes J.A., Cortassa S., Kaushik S., Gonzalez-Freire M., Patel B. (2016). Effects of Sex, Strain, and Energy Intake on Hallmarks of Aging in Mice. Cell Metab..

[53] Selman C., Lingard S., Choudhury A.I., Batterham R.L., Claret M., Clements M., Ramadani F., Okkenhaug K., Schuster E., Blanc E. (2007). Evidence for lifespan extension and delayed age-related biomarkers in insulin receptor substrate 1 null mice. FASEB J..

[54] Sun N., Youle R.J., Finkel T. (2016). The Mitochondrial Basis of Aging. Mol. Cell.

[55] Von Zglinicki T. (2002). Oxidative stress shortens telomeres. Trends Biochem. Sci..

[56] Moiseeva O., Bourdeau V., Roux A., Deschênes-Simard X., Ferbeyre G. (2009). Mitochondrial Dysfunction Contributes to Oncogene-Induced Senescence. Mol. Cell. Biol..

[57] Baines H.L., Turnbull U.M., Greaves L.C. (2014). Human stem cell aging: Do mitochondrial DNA mutations have a causal role?. Aging Cell.

[58] Marioni R.E., Shah S., McRae A.F., Chen B.H., Colicino E., Harris S.E., Gibson J., Henders A.K., Redmond P., Cox S.R. (2015). DNA methylation age of blood predicts all-cause mortality in later life. Genome Biol..

[59] Baker D.J., Dawlaty M.M., Wijshake T., Jeganathan K., Malureanu L., Van Ree J.H., Crespo-Diaz R., Reyes S., Seaburg L., Shapiro V. (2013). Increased expression of BubR1 protects against aneuploidy and cancer and extends healthy lifespan. Nat. Cell Biol..

[60] Pereira M., Ribas de Farias Costa P., Pereira E.M., Russoni de Lima Lago I., Oliveira A.M. (2021). Does vitamin D deficiency increase the risk of obesity in adults and the elderly? A systematic review of prospective cohort studies. Public Health.

[61] VanLint S. (2013). Vitamin D and Obesity. Nutrients.

[62] Walsh J., Bowles S., Evans A.L. (2017). Vitamin D in obesity. Curr. Opin. Endocrinol. Diabetes Obes..

[63] De Oliveira L.F., De Azevedo L.G., da Mota Santana J., De Sales L.P.C., Pereira-Santos M. (2019). Obesity and overweight decreases the effect of vitamin D supplementation in adults: Systematic review and meta-analysis of randomized controlled trials. Rev. Endocr. Metab. Disord..

[64] Kull M., Kallikorm R., Lember M. (2009). Body mass index determines sunbathing habits: Implications on vitamin D levels. Intern. Med. J..

[65] Rock C.L., Emond J.A., Flatt S.W., Heath D.D., Karanja N., Pakiz B., Sherwood N.E., Thomson C.A. (2012). Weight Loss Is Associated With Increased Serum 25-Hydroxyvitamin D in Overweight or Obese Women. Obesity.

[66] Gangloff A., Bergeron J., Pelletier-Beaumont E., Nazare J.-A., Smith J., Borel A.-L., Lemieux I., Tremblay A., Poirier P., Alméras N. (2015). Effect of adipose tissue volume loss on circulating 25-hydroxyvitamin D levels: Results from a 1-year lifestyle intervention in viscerally obese men. Int. J. Obes..

[67] Calton E.K., Keane K.N., Newsholme P., Soares M.J. (2015). The Impact of Vitamin D Levels on Inflammatory Status: A Systematic Review of Immune Cell Studies. PLoS ONE.

[68] Boutens L., Stienstra R. (2016). Adipose tissue macrophages: Going off track during obesity. Diabetologia.

[69] Matsuda M., Shimomura I. (2014). Roles of oxidative stress, adiponectin, and nuclear hormone receptors in obesity-associated insulin resistance and cardiovascular risk. Horm. Mol. Biol. Clin. Investig..

[70] Boucher B.J. (2007). “Inverse correlation between serum free IGF-1 and IGFBP-3 levels and blood pressure in patients affected with type 1 diabetes” by Capoluongo et al. Cytokine.

[71] Moore W.T., Bowser S.M., Fausnacht D.W., Staley L.L., Suh K.-S., Liu N. (2015). Beta Cell Function and the Nutritional State: Dietary Factors that Influence Insulin Secretion. Curr. Diabetes Rep..

[72] Leung P.S. (2016). The Potential Protective Action of Vitamin D in Hepatic Insulin Resistance and Pancreatic Islet Dysfunction in Type 2 Diabetes Mellitus. Nutrients.

[73] Ji L., Gupta M., Feldman B.J. (2016). Vitamin D Regulates Fatty Acid Composition in Subcutaneous Adipose Tissue Through Elovl3. Endocrinology.

[74] Li Y.C., Kong J., Wei M., Chen Z.-F., Liu S.Q., Cao L.-P. (2002). 1,25-Dihydroxyvitamin D3 is a negative endocrine regulator of the renin-angiotensin system. J. Clin. Investig..

[75] Burton D.G.A., Faragher R.G.A. (2018). Obesity and type-2 diabetes as inducers of premature cellular senescence and ageing. Biogerontology.

[76] Minamino T., Orimo M., Shimizu I., Kunieda T., Yokoyama M., Ito T., Nojima A., Nabetani A., Oike Y., Matsubara H. (2009). A crucial role for adipose tissue p53 in the regulation of insulin resistance. Nat. Med..

[77] Scott S.H., Bahnson B.J. (2011). Senescence marker protein 30: Functional and structural insights to its unknown physiological function. Biomol. Concepts.

[78] Bima A., Mahdi A., Al Fayez F., Khawaja T., El-Khair S.A., Elsamanoudy A. (2021). Cellular Senescence and Vitamin D Deficiency Play a Role in the Pathogenesis of Obesity-Associated Subclinical Atherosclerosis: Study of the Potential Protective Role of Vitamin D Supplementation. Cells.

[79] Chen Y.-W., Harris R.A., Hatahet Z., Chou K.-M. (2015). Ablation of XP-V gene causes adipose tissue senescence and metabolic abnormalities. Proc. Natl. Acad. Sci. USA.

[80] Monickaraj F., Aravind S., Nandhini P., Prabu P., Sathishkumar C., Mohan V., Balasubramanyam M. (2013). Accelerated fat cell aging links oxidative stress and insulin resistance in adipocytes. J. Biosci..

[81] Mitterberger M.C., Lechner S., Mattesich M., Zwerschke W. (2013). Adipogenic Differentiation Is Impaired in Replicative Senescent Human Subcutaneous Adipose-Derived Stromal/Progenitor Cells. J. Gerontol. Ser. A Biol. Sci. Med. Sci..

[82] Zhao M., Chen X. (2015). Effect of lipopolysaccharides on adipogenic potential and premature senescence of adipocyte progenitors. Am. J. Physiol. Endocrinol. Metab..

[83] Xu M., Palmer A., Ding H., Weivoda M.M., Pirtskhalava T., White T., Sepe A., Johnson K., Stout M.B., Giorgadze N. (2015). Targeting senescent cells enhances adipogenesis and metabolic function in old age. eLife.

[84] Nowak J., Hudzik B., Jagielski P., Kulik-Kupka K., Danikiewicz A., Zubelewicz-Szkodzińska B. (2021). Lack of Seasonal Variations in Vitamin D Concentrations among Hospitalized Elderly Patients. Int. J. Environ. Res. Public Health.

[85] Gallagher J.C. (2013). Vitamin D and Aging. Endocrinol. Metab. Clin. N. Am..

[86] Chen L., Yang R., Qiao W., Zhang W., Chen J., Mao L., Goltzman D., Miao D. (2019). 1,25-Dihydroxyvitamin D exerts an antiaging role by activation of Nrf2-antioxidant signaling and inactivation of p16/p53-senescence signaling. Aging Cell.

[87] Lewis K.N., Mele J., Hayes J.D., Buffenstein R. (2010). Nrf2, a Guardian of Healthspan and Gatekeeper of Species Longevity. Integr. Comp. Biol..

[88] Nakai K., Fujii H., Kono K., Goto S., Kitazawa R., Kitazawa S., Hirata M., Shinohara M., Fukagawa M., Nishi S. (2013). Vitamin D Activates the Nrf2-Keap1 Antioxidant Pathway and Ameliorates Nephropathy in Diabetic Rats. Am. J. Hypertens..

[89] Forster R.E., Jurutka P.W., Hsieh J.-C., Haussler C.A., Lowmiller C.L., Kaneko I., Haussler M.R., Whitfield G.K. (2011). Vitamin D receptor controls expression of the anti-aging klotho gene in mouse and human renal cells. Biochem. Biophys. Res. Commun..

[90] Kuro O.M. (2009). Klotho and aging. Biochim. Biophys. Acta.

[91] Berridge M.J. (2017). Vitamin D deficiency accelerates ageing and age-related diseases: A novel hypothesis. J. Physiol..

[92] Zarei M., Zarezadeh M., Kalajahi F.H., Javanbakht M.H. (2020). The Relationship Between Vitamin D and Telomere/Telomerase: A comprehensive review. J. Frailty Aging.

[93] Hoffecker B.M., Raffield L., Kamen D.L., Nowling T.K. (2013). Systemic Lupus Erythematosus and Vitamin D Deficiency Are Associated with Shorter Telomere Length among African Americans: A Case-Control Study. PLoS ONE.

[94] Liu J.J., Prescott J., Giovannucci E., Hankinson S.E., Rosner B., Han J., De Vivo I. (2013). Plasma Vitamin D Biomarkers and Leukocyte Telomere Length. Am. J. Epidemiol..

[95] Richards J.B., Valdes A., Gardner J.P., Paximadas D., Kimura M., Nessa A., Lu X., Surdulescu G.L., Swaminathan R., Spector T.D. (2007). Higher serum vitamin D concentrations are associated with longer leukocyte telomere length in women. Am. J. Clin. Nutr..

[96] Aziz M., Yadav K.S. (2016). Pathogenesis of Atherosclerosis A Review. Med. Clin. Rev..

[97] Weber C., Noels H. (2011). Atherosclerosis: Current pathogenesis and therapeutic options. Nat. Med..

[98] Singh S.S., Pilkerton C.S., Shrader C.D., Frisbee S.J. (2018). Subclinical atherosclerosis, cardiovascular health, and disease risk: Is there a case for the Cardiovascular Health Index in the primary prevention population?. BMC Public Health.

[99] Lusis A.J. (2012). Genetics of atherosclerosis. Trends Genet..

[100] Blaha M.J., Rivera J.J., Budoff M.J., Blankstein R., Agatston A., O’Leary D.H., Cushman M., Lakoski S., Criqui M.H., Szklo M. (2011). Association Between Obesity, High-Sensitivity C-Reactive Protein ≥2 mg/L, and Subclinical Atherosclerosis: Implications of JUPITER from the Multi-Ethnic Study of Atherosclerosis. Arter. Thromb. Vasc. Biol..

[101] Fox C.S., Massaro J.M., Hoffmann U., Pou K.M., Maurovich-Horvat P., Liu C.-Y., Vasan R.S., Murabito J., Meigs J.B., Cupples L.A. (2007). Abdominal Visceral and Subcutaneous Adipose Tissue Compartments. Circulation.

[102] Qasim A., Mehta N.N., Tadesse M.G., Wolfe M.L., Rhodes T., Girman C., Reilly M.P. (2008). Adipokines, Insulin Resistance, and Coronary Artery Calcification. J. Am. Coll. Cardiol..

[103] Ouchi N., Kihara S., Arita Y., Okamoto Y., Maeda K., Kuriyama H., Hotta K., Nishida M., Takahashi M., Muraguchi M. (2000). Adiponectin, an Adipocyte-Derived Plasma Protein, Inhibits Endothelial NF-κB Signaling Through a cAMP-Dependent Pathway. Circulation.

[104] Parhami F., Tintut Y., Ballard A., Fogelman A.M., Demer L.L. (2001). Leptin Enhances the Calcification of Vascular Cells. Circ. Res..

[105] Rossello X., Fuster V., Oliva B., Sanz J., Friera L.A.F., López-Melgar B., Mendiguren J.M., Pezzi E.L.-, Bueno H., Fernández-Ortiz A. (2020). Association Between Body Size Phenotypes and Subclinical Atherosclerosis. J. Clin. Endocrinol. Metab..

[106] Kim S., Kyung C., Park J.S., Lee S.-P., Kim H.K., Ahn C.W., Kim K.R., Kang S. (2015). Normal-weight obesity is associated with increased risk of subclinical atherosclerosis. Cardiovasc. Diabetol..

[107] Akin F., Ayça B., Köse N., Duran M., Sarı M., Uysal O.K., Karakukcu C., Arinc H., Covic A., Goldsmith D. (2012). Serum Vitamin D Levels Are Independently Associated With Severity of Coronary Artery Disease. J. Investig. Med..

[108] Al Mheid I., Patel R., Murrow J., Morris A., Aznaouridis K., Rahman A., Fike L., Kavtaradze N., Ahmed Y., Uphoff I. (2011). Vitamin D status is associated with arterial stiffness and vascular dysfunction in healthy humans. J. Am. Coll. Cardiol..

[109] Satilmis S., Celik O., Biyik I., Ozturk D., Celik K., Akın F., Ayca B., Yalcin B., Dagdelen S. (2015). Association between serum vitamin D levels and subclinical coronary atherosclerosis and plaque burden/composition in young adult population. Bosn. J. Basic Med. Sci..

[110] Siasos G., Tousoulis D., Oikonomou E., Maniatis K., Kioufis S., Kokkou E., Miliou A., Zaromitidou M., Kassi E., Stefanadis C. (2013). Vitamin D serum levels are associated with cardiovascular outcome in coronary artery disease. Int. J. Cardiol..

[111] Tarcin O., Yavuz D.G., Ozben B., Telli A., Ogunc A.V., Yuksel M., Toprak A., Yazici D., Sancak S., Deyneli O. (2009). Effect of Vitamin D Deficiency and Replacement on Endothelial Function in Asymptomatic Subjects. J. Clin. Endocrinol. Metab..

[112] Lai H., Fishman E., Gerstenblith G., Brinker J.A., Tong W., Bhatia S., Detrick B., Lai S. (2012). Vitamin D deficiency is associated with significant coronary stenoses in asymptomatic African American chronic cocaine users. Int. J. Cardiol..

[113] Lee S., Ahuja V., Masaki K., Evans R.W., Barinas-Mitchell E., Ueshima H., Shin C., Choo J., Hassen L., Edmundowicz D. (2016). A Significant Positive Association of Vitamin D Deficiency with Coronary Artery Calcification among Middle-aged Men: For the ERA JUMP Study. J. Am. Coll. Nutr..

[114] Pittas A.G., Lau J., Hu F.B., Dawson-Hughes B. (2007). The Role of Vitamin D and Calcium in Type 2 Diabetes. A Systematic Review and Meta-Analysis. J. Clin. Endocrinol. Metab..

[115] Oh J., Weng S., Felton S.K., Bhandare S., Riek A., Butler B., Proctor B.M., Petty M., Chen Z., Schechtman K.B. (2009). 1,25(OH) 2 Vitamin D Inhibits Foam Cell Formation and Suppresses Macrophage Cholesterol Uptake in Patients With Type 2 Diabetes Mellitus. Circulation.

[116] Murr C., Pilz S., Grammer T.B., Kleber M., Meinitzer A., Boehm B.O., März W., Fuchs D. (2012). Vitamin D deficiency parallels inflammation and immune activation, the Ludwigshafen Risk and Cardiovascular Health (LURIC) study. Clin. Chem. Lab. Med..

[117] Shea M.K., Booth S.L., Massaro J.M., Jacques P.F., D’Agostino R.B., Dawson-Hughes B., Ordovas J.M., O’Donnell C.J., Kathiresan S., Keaney J.F. (2008). Vitamin K and Vitamin D Status: Associations with Inflammatory Markers in the Framingham Offspring Study. Am. J. Epidemiol..

[118] Nilsson P.M., Boutouyrie P., Cunha P., Kotsis V., Narkiewicz K., Parati G., Rietzschel E., Scuteri A., Laurent S. (2013). Early vascular ageing in translation. J. Hypertens..

[119] Iurciuc S., Cimpean A.M., Mitu F., Heredea R., Iurciuc M. (2017). Vascular aging and subclinical atherosclerosis: Why such a “never ending” and challenging story in cardiology?. Clin. Interv. Aging.

[120] Nilsson P.M., Boutouyrie P., Laurent S. (2009). Vascular Aging. Hypertension.

[121] Fernández-Alvira J.M., Fuster V., Dorado B., Soberón N., Flores I., Gallardo M., Pocock S., Blasco M.A., Andrés V. (2016). Short Telomere Load, Telomere Length, and Subclinical Atherosclerosis. J. Am. Coll. Cardiol..

[122] Sahin E., Colla S., Liesa M., Moslehi J., Muller F., Guo M., Cooper M., Kotton D., Fabian A.J., Walkey C. (2011). Telomere dysfunction induces metabolic and mitochondrial compromise. Nature.

[123] Hunt S.C., Kark J.D., Aviv A. (2015). Association Between Shortened Leukocyte Telomere Length and Cardio-Metabolic Outcomes. Circ. Cardiovasc. Genet..

[124] Langlois M.R., Rietzschel E.R., De Buyzere M.L., De Bacquer D., Bekaert S., Blaton V., De Backer G.G., Gillebert T.C. (2008). Femoral Plaques Confound the Association of Circulating Oxidized Low-Density Lipoprotein With Carotid Atherosclerosis in a General Population Aged 35 to 55 Years. Arter. Thromb. Vasc. Biol..

[125] Godoy-Matos A.F., Júnior W.S.S., Valerio C.M. (2020). NAFLD as a continuum: From obesity to metabolic syndrome and diabetes. Diabetol. Metab. Syndr..

[126] Al-Ghamdi H.A., Al Fayez F.F., Bima A.I., Khawaji T.M., Elsamanoudy A.Z. (2021). Study of Cellular Senescence and Vitamin D Deficiency in Nonalcoholic Fatty Liver Disease and The Potential Protective Effect of Vitamin D Supplementation. J. Clin. Exp. Hepatol..

[127] Mota M., Banini B.A., Cazanave S.C., Sanyal A.J. (2016). Molecular mechanisms of lipotoxicity and glucotoxicity in nonalcoholic fatty liver disease. Metab. Clin. Exp..

[128] Kitson M.T., Roberts S.K. (2012). D-livering the message: The importance of vitamin D status in chronic liver disease. J. Hepatol..

[129] Roth C.L., Elfers C.T., Figlewicz D.P., Melhorn S.J., Morton G.J., Hoofnagle A., Yeh M.M., Nelson J.E., Kowdley K.V. (2012). Vitamin D deficiency in obese rats exacerbates nonalcoholic fatty liver disease and increases hepatic resistin and toll-like receptor activation. Hepatology.

[130] Liangpunsakul S., Chalasani N. (2011). Serum Vitamin D Concentrations and Unexplained Elevation in ALT Among US Adults. Dig. Dis. Sci..

[131] Cordeiro A., Pereira S., Saboya C., Ramalho A. (2017). Relationship between Nonalcoholic Fatty Liver Disease and Vitamin D Nutritional Status in Extreme Obesity. Can. J. Gastroenterol. Hepatol..

[132] Nakano T., Cheng Y.-F., Lai C.-Y., Hsu L.-W., Chang Y.-C., Deng J.-Y., Huang Y.-Z., Honda H., Chen K.-D., Wang C.-C. (2011). Impact of artificial sunlight therapy on the progress of non-alcoholic fatty liver disease in rats. J. Hepatol..

[133] Arai T., Atsukawa M., Tsubota A., Koeda M., Yoshida Y., Okubo T., Nakagawa A., Itokawa N., Kondo C., Nakatsuka K. (2019). Association of vitamin D levels and vitamin D-related gene polymorphisms with liver fibrosis in patients with biopsyproven nonalcoholic fatty liver disease. Dig. Liver Dis..

[134] Wang M., Zhang R., Wang M., Zhang L., Ding Y., Tang Z., Wang H., Zhang W., Chen Y., Wang J. (2021). Genetic Polymorphism of Vitamin D Family Genes CYP2R1, CYP24A1, and CYP27B1 Are Associated With a High Risk of Non-alcoholic Fatty Liver Disease: A Case-Control Study. Front. Genet..

[135] Jiang X., O’Reilly P., Aschard H., Hsu Y.-H., Richards J.B., Dupuis J., Ingelsson E., Karasik D., Pilz S., Berry D. (2018). Genome-wide association study in 79,366 European-ancestry individuals informs the genetic architecture of 25-hydroxyvitamin D levels. Nat. Commun..

[136] Qian P., Cao X., Xu X., Duan M., Zhang Q., Huang G. (2020). Contribution of CYP24A1 variants in coronary heart disease among the Chinese population. Lipids Health Dis..

[137] Papatheodoridi A., Chrysavgis L., Koutsilieris M., Chatzigeorgiou A. (2020). The Role of Senescence in the Development of Nonalcoholic Fatty Liver Disease and Progression to Nonalcoholic Steatohepatitis. Hepatology.

[138] Zhang X., Zhou D., Strakovsky R., Zhang Y., Pan Y.-X. (2012). Hepatic cellular senescence pathway genes are induced through histone modifications in a diet-induced obese rat model. Am. J. Physiol. Liver Physiol..

[139] Ogrodnik M., Miwa S., Tchkonia T., Tiniakos D., Wilson C.L., Lahat A., Day C.P., Burt A., Palmer A., Anstee Q.M. (2017). Cellular senescence drives age-dependent hepatic steatosis. Nat. Commun..

[140] Nguyen P., Valanejad L., Cast A., Wright M., Garcia J.M., El-Serag H.B., Karns R., Timchenko N.A. (2018). Elimination of Age-Associated Hepatic Steatosis and Correction of Aging Phenotype by Inhibition of cdk4-C/EBPα-p300 Axis. Cell Rep..

[141] Aravinthan A., Scarpini C.G., Tachtatzis P., Verma S., Penrhyn-Lowe S., Harvey R., Davies S.E., Allison M., Coleman N., Alexander G. (2013). Hepatocyte senescence predicts progression in non-alcohol-related fatty liver disease. J. Hepatol..

[142] Aravinthan A., Mells G., Allison M., Leathart J., Kotronen A., Yki-Järvinen H., Daly A.K., Day C.P., Anstee Q.M., Alexander G. (2014). Gene polymorphisms of cellular senescence marker p21 and disease progression in non-alcohol-related fatty liver disease. Cell Cycle.

[143] Hardy T., Zeybel M., Day C.P., Dipper C., Masson S., McPherson S., Henderson E., Tiniakos D., White S., French J. (2017). Plasma DNA methylation: A potential biomarker for stratification of liver fibrosis in non-alcoholic fatty liver disease. Gut.

[144] Byrne C.D., Targher G. (2015). NAFLD: A multisystem disease. J. Hepatol..

[145] VanWagner L.B. (2018). New insights into NAFLD and subclinical coronary atherosclerosis. J. Hepatol..

[146] Wójcik-Cichy K., Koślińska-Berkan E., Piekarska A. (2018). The influence of NAFLD on the risk of atherosclerosis and cardiovascular diseases. Clin. Exp. Hepatol..

[147] Zhou Y., Zhou X., Wu S., Fan D., Van Poucke S., Chen Y., Fu S., Zheng M. (2018). Nonalcoholic fatty liver disease contributes to subclinical atherosclerosis: A systematic review and meta-analysis. Hepatol. Commun..

